# Cerebral infarction as initial presentation in stress cardiomyopathy: Case report and literature review

**DOI:** 10.1097/MD.0000000000010804

**Published:** 2018-05-18

**Authors:** Qiongying Wang, Heng Yu, Cheng Jiang, Runmin Sun, Miaomiao Qi, Shougang Sun, Guangli Xu, Hongbin Cai, Zhenchang Zhang, Feng Zhao, Xiaoqing Kou, Jing Yu, Feng Bai

**Affiliations:** a Department of Cardiology; b Department of Neurology, Lanzhou University Second Hospital, Lanzhou, Gansu, P.R. China.

**Keywords:** cerebral infarction, left ventricular thrombus, stress cardiomyopathy

## Abstract

**Rationale:**

The typical symptoms of stress cardiomyopathy include sudden-onset chest pain and breathlessness or collapse as well as classical symptoms of cardiovascular disease; however, rare reports have described nervous system symptoms as the initial manifestation. Here, we report the case of a young man who presented with a large cerebral infarction as the main clinical symptom of stress cardiomyopathy to increase recognition of the disease.

**Patient concerns:**

A 28-year-old man was admitted to our hospital for sudden-onset weakness of the right limbs and unconsciousness for 1 day. Ten days prior, he began consuming copious amounts of alcohol (500 mL/day) secondary to reactive depression.

**Diagnoses:**

Imaging revealed a left internal carotid artery occlusion as assessed by carotid artery ultrasonography. Brain magnetic resonance imaging/magnetic resonance angiography showed new large left cerebral infarction complicated by a reperfusion injury. Moreover, cardiac ultrasonography showed decreased motion of the left ventricular apex, a 3.7 cm^2^ mural thrombus in the ventricular apex. The results of coronary and renal artery angiography did not reveal any significant epicardial coronary disease with thrombolysis in the myocardial infarction grade 3 in any of the coronary arteries.

**Interventions:**

The patient was administered antiplatelet, anticoagulation, antihypertension, antibiotic, and neurotrophic therapies.

**Outcomes:**

The symptoms of cerebral infarction improved significantly after 12 days of admission. Cardiac ultrasonography showed that the wall movement of the left ventricular apex had recovered fully and the mural thrombus resolved completely.

**Lessons:**

Patients with stress cardiomyopathy exhibit various clinical manifestations and characteristics. On the basis of our in-depth understanding of stress cardiomyopathy, clinicians should diagnose early and develop reasonable and effective therapies to prevent the harmful effects of related complications.

## 1 Introduction

Stress cardiomyopathy, also known as Takotsubo cardiomyopathy, left ventricular apical ballooning syndrome, or broken heart syndrome, is usually characterized by transient left ventricular systolic dysfunction without obstructive coronary disease. Most patients recover completely, the recurrence rate is ≤10%,^[1]^ and patients might exhibit severe cardiovascular complications during the disease.^[2]^ Nevertheless, almost no reports describe cases of nervous system symptoms as the initial manifestations. Here, we report a case of a young man who presented with a large cerebral infarction caused by a left ventricular thrombus. As the clinical symptoms and expression of stress cardiomyopathy vary, clinicians should notice any abnormalities during the patient work-up.

This case report was approved by the ethics committee of the Lanzhou University Second Hospital, Lanzhou, China. Informed consent was obtained from the patient for the publication of this case report.

## 2 Case report

A 28-year-old man presented to Lanzhou University Second Hospital in China with sudden-onset weakness of the right limbs and unconsciousness for 1 day. Ten days prior, he had started consuming copious amounts of alcohol (500 mL/day) secondary to reactive depression. One day before admission, he presented with weakness of the right limbs (0/6 muscle strength of the right upper and lower limbs), unconsciousness, and a lack of response to verbal stimuli. Subsequently, he was admitted to a local county hospital for treatment. A head computed tomography (CT) scan revealed a low-density lesion in the left temporal/parietal lobes. He was initially diagnosed with cerebral infarction and then transferred to Lanzhou University Second Hospital for further management.

**Figure 1:**
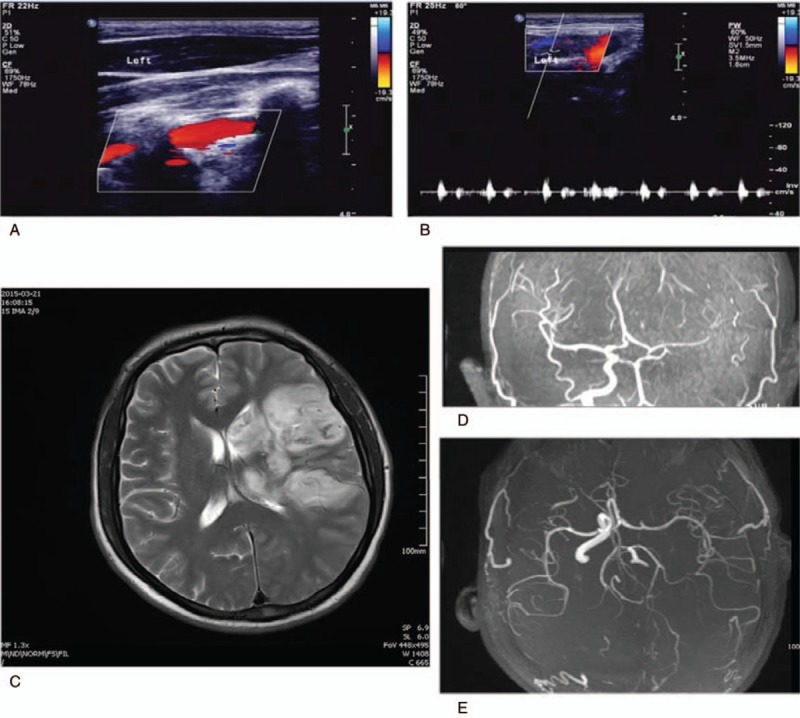
Carotid ultrasonography and cranial magnetic resonance imaging (MRI). (A) Left carotid artery was occluded and lacked blood flow; (B) Blood flow signals not detected in the left carotid artery; (C) Cranial MRI showing a new large cerebral infarction in the left hemisphere; (D, E) The left internal carotid artery is occluded, right internal carotid artery supplies compensatory blood to the left anterior cerebral artery and middle cerebral artery through the anterior communicating artery, and distal branches of the left middle cerebral artery are poorly visualized.

**Figure 2:**
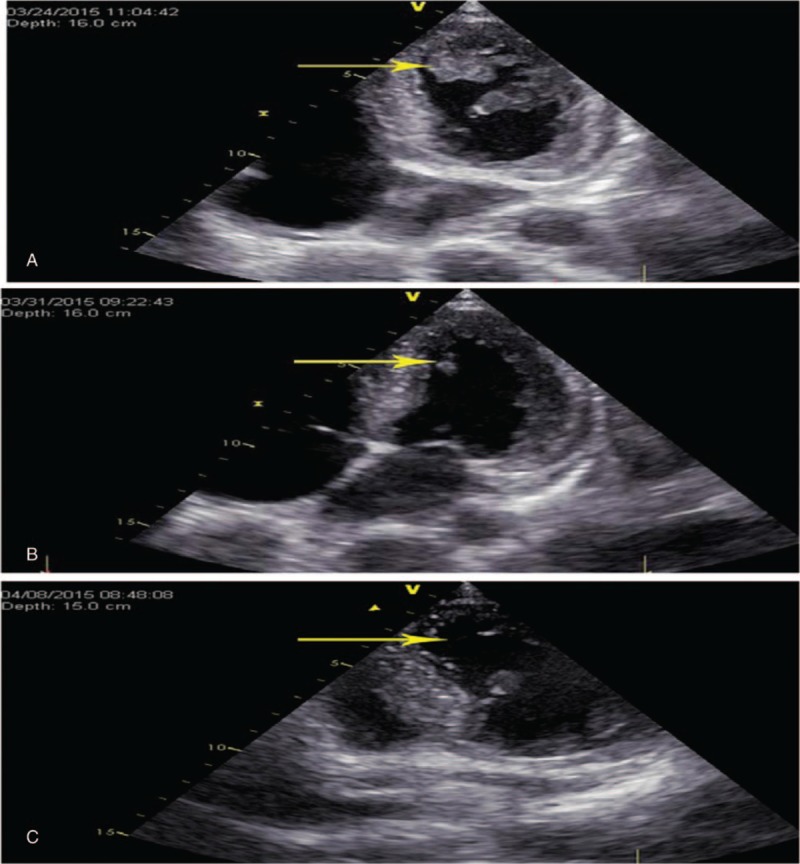
Changes in the apical thrombus visible on echocardiography.

Upon regaining consciousness, he complained of dizziness, headache, nausea, and nonejection vomiting but no foaming at the mouth or twitching of the limbs. Before this event, the patient was healthy and had no family history of seizures or stroke. The patient had risk factors for stroke including a 10-year smoking history and ethanol use. The physical examination at the time of admission revealed a body temperature of 38.5°C, pulse of 105 beats/min, respiratory rate of 19 times/min, and blood pressure of 152/106 mm Hg. Overall, he was somnolent. Auscultation of the lungs detected rough breath sounds but no rhonchi or moist rales. A cardiac examination should a nondisplaced point of maximal impulse with regular tachycardia. There were no murmurs, rubs, or gallops. A neurological examination revealed 0/6 muscle strength of the right upper and lower limbs. A laboratory examination revealed total white blood cells of 16.31 × 10^9^/L with 90% neutrophils. Biochemical tests revealed elevated myocardial enzymes, including creatine kinase 566 U, creatine kinase isoenzymes 78 U, and cardiac troponin I 0.23 μg/L. Electrocardiography (ECG) showed sinus rhythm, P-wave terminal force in lead V1 = -0.04 ms, QS pattern in leads V1–V3, and T-wave inversion in II, III, avF, and V4-V6. Imaging revealed a left internal carotid artery occlusion as assessed by carotid artery ultrasonography (Fig. 1A, B),low velocity and low pulsation in the left cerebral hemisphere, an opened anterior communicating branch, and a compensatory increase in blood flow velocity of the right anterior cerebral artery as evidenced by transcranial Doppler. Brain magnetic resonance imaging/magnetic resonance angiography (MRI/MRA) showed new large left cerebral infarction complicated by the reperfusion injury. New infarcts were seen in the right parietal lobe and the genu of the corpus callosum as well as a left internal carotid artery occlusion (Fig. 1C, D). Moreover, cardiac ultrasonography showed decreased motion of the left ventricular apex, a 3.7 cm^2^ mural thrombus in the ventricular apex, and left ventricular hypertrophy. The left ventricular ejection fraction (LVEF) was 50% (Fig. 2A). The results of coronary and renal artery angiography performed 15 days after admission did not reveal any significant epicardial coronary disease with thrombolysis in the myocardial infarction (TIMI) grade 3 in all coronary arteries.

Considering the ECG changes, evidence of myocyte injury (biomarker elevation), normal coronary angiography and echocardiography findings for wall motion abnormalities, and his medical history, the patient was diagnosed with stress cardiomyopathy. Accordingly, he was administered antiplatelet (aspirin 100 mg orally once a day), anticoagulation (nadroparin calcium 0·4 mL, two times/day and warfarin 4.5 mg orally once a day), antihypertension (irbesartan 150 mg orally once a day, metoprolol 90 mg orally once a day), antibiotic (levofloxacin 0.2 g intravenous drip, two times/day for 5 days), hydration (mannitol 125 mL intravenous drip, two times/day for 7 days), and neurotrophic (butylphthalide 100 mL intravenous drip, two times/day) therapies. Consequently, the symptoms of cerebral infarction improved significantly 12 days after admission, and the muscle strength of the right upper and lower limb was recovered to 4/6. Repeat cardiac ultrasonography showed that the wall movement of the left ventricular apex had recovered fully, left ventricular mural thrombus decreased to 0.6 cm^2^, and LVEF was 72%. At 20 days, cardiac ultrasonography showed complete resolution of the mural thrombus (Figs. 2B, C). After a schedule was created for subsequent treatment and follow-up, the patient was discharged from the hospital. To prevent further thrombotic events, oral warfarin was administered after discharged 6 months (prothrombin time international normalized ratio, 2–3). Standard antihypertension therapy consisting of irbesartan and metoprolol was also administered. The 6-month follow-up showed that the muscle strength had recovered to 5/6. The patient is now in a stable condition, attends follow-up appointments every 6 months, and continues treatment with aspirin, irbesartan, and metoprolol.

## 3 Discussion

The most common sources of emboli are the heart and limb blood vessels. Cardiac sources of emboli are common in rheumatic heart disease, atrial fibrillation, subacute bacterial endocarditis, myocardial infarction, dilated cardiomyopathy, and noncompaction of the ventricular myocardium. Among them, the incidence of cerebral embolism within 1 month after acute myocardial infarction (AMI) was about 2.5%, and most of the thrombus originated from the left ventricle.^[3]^ A recent follow-up study showed that the incidence of cerebral embolism in patients with left ventricular thrombi within 2 years was 10% to 15%, most of which occurred within the first 10 days of thrombosis formation.^[4]^

The primary manifestation in this patient was cerebral infarction. The results of echocardiography performed on day 5 after onset showed that the LVEF was 50%, the myocardial motion of the left ventricular apex was decreased, and there was a 3.7 cm^2^ block-like moderate-intensity echogenic mass was seen in the left ventricle. Carotid artery ultrasonography showed left internal carotid artery occlusion. Brain MRI/MRA showed a new large cerebral infarction complicated by the reperfusion injury in the left hemisphere, new infarcts in the right parietal lobe and the genu of the corpus callosum, and a left internal carotid artery occlusion. Thus, the cerebral embolism in this young man originated from the left ventricle.

The origin of the ventricular mural thrombus was most likely secondary to Takotsubo myocarditis. Myocardial injury markers were elevated in the absence of typical clinical manifestations of acute coronary syndrome (ACS) such as chest pain, chest tightness, and precordial discomfort. ECG showed the QS pattern in leads V1–V3 as well as T-wave inversion in leads II, III, avF, and V4–V6. However, coronary angiography did not reveal any abnormalities in the coronary arteries and TIMI grade 3 blood flow. Therefore, myocardial infarction caused by acute coronary artery occlusion could be excluded.

Key characteristics for the diagnosis of Takotsubo cardiomyopathy (Mayo Clinic Criteria 2014) include wall motion abnormalities (generally extending beyond a typical coronary distribution); and evidence of myocyte injury, including biomarker elevation and ECG changes. Supportive clinical features include postmenopausal woman or preceding stressor. Key exclusions include coronary obstruction sufficient to explain the wall motion abnormalities (myocarditis and/or pheochromocytoma).^[5]^ Combined with the patient's medical history and the myocardial injury marker, coronary angiography, and echocardiography results, stress cardiomyopathy can be considered the appropriate diagnosis for the patient's symptoms and disease outcome during hospitalization.

## 4 Limitations

With the strong emotional stress medical history, ECG changes, evidence biomarker elevation, thrombus in the ventricular apex, and regional wall motion abnormality, we should consider stress cardiomyopathy earlier, assess the coronary artery, and perform left ventriculography using a catheter on radiography. Frequently described triggers of stress cardiomyopathy include sudden strong emotional or physical stress as in this patient, for whom divorce was the main cause of this onset. We focused only on the treatment of the patient's brain injury, ventricular thrombus, and blood pressure and neglected psychotherapy. As a result, the patient was admitted to the emergency center again for swallowing pesticides on the same day the next year.

Although there are still no official guidelines for the treatment of stress cardiomyopathy, psychotherapy is important for such patients.

## 5 Literature review of stress cardiomyopathy

As Dote et al^[6]^ first described transient left ventricular apical ballooning syndrome in 1991, our understanding and diagnosis of stress cardiomyopathy have continuously improved. Most cases of this disease present with chest pain as the initial symptom combined with ST-segment and Q-wave changes in the ECG without abnormal occlusion of the coronary arteries.

In another study, approximately 2% of patients with ACS as the primary clinical manifestation were finally diagnosed with stress cardiomyopathy.^[7]^ Chest pain or precordial discomfort was the most common primary clinical manifestation of this disease, and it could also manifest as palpitations, nausea, vomiting, syncope, and dyspnea. Acute pulmonary edema, cardiogenic shock, respiratory failure, and other manifestations resembling ACS might occur in the acute phase of the disease.

A study from Hiroshima City Hospital^[8]^ found that 5 of 95 patients were diagnosed with stress cardiomyopathy combined with left ventricular apical thrombus at an incidence of 5.3%. Two of 5 patients with left ventricular apical thrombus had a mural thrombus, while the other 3 increased thrombus. The apical thrombus in 4 patients disappeared after anticoagulant therapy, while 1 patient had a cerebral infarction.

Formation of the left ventricular apical thrombus was one of the complications of anterior myocardial infarction, and its incidence was 30% to 43% before the AMI reperfusion era and 8% to 20% after the reperfusion era. The thrombosis in the left ventricle was associated with left ventricular wall motion abnormalities, decreased left ventricular function, and increased left ventricular volume in patients combined with clinical heart failure. Stress cardiomyopathy often occurred with transient left ventricular apical dilatation and left ventricular apical aneurysm, which was the primary cause of the formation of left ventricular apical thrombus.^[9,10]^ In Takotsubo cardiomyopathy, transient left ventricular (LV) apical aneurysm always occurs during the early phase, which might be the most important factor related to LV apical thrombosis. In addition to local hemostasis caused by LV apical aneurysm, several factors may be responsible for LV apical thrombosis, such as endocardial injury with local exposure or release of thrombogenic substances.

Stress cardiomyopathy usually manifests in various forms as sites of ventricular dysfunction; however, its precise pathogenic factors have not yet been fully elucidated. The syndrome is speculated to be due to the following reasons: myocardial toxicity caused by elevated catecholamine levels^[5]^; the myocardial stunning caused by excessive activation of the central and peripheral sympathetic nerves altered the neurotransmitter levels^[11]^; abnormal vascular structure of the coronary arteries^[12]^; coronary microvascular spasm or dysfunction^[13]^; decreased estradiol levels^[14]^; left ventricular outflow tract obstruction^[15]^; virus-induced myocardial inflammation^[16]^; and fatty-acid metabolism disorder.^[17]^

Some investigators reported that the in-hospital mortality of this disease was only 1.7% and that 95% patients could recover completely, while only a few relapsed.^[18]^ The pathological process of stress cardiomyopathy was temporary and reversible, and its clinical prognosis was better than that of AMI; however, it could still lead to sudden death in patients without organic heart disease. The incidence of severe complications was high during the acute phase of the disease.

Echocardiography and cardiac magnetic resonance imaging found typical wall motion abnormalities, left ventricular thrombosis, and left ventricular systolic dysfunction. Echocardiography was often performed before coronary angiography and left ventriculography^[19–22]^ in critically ill patients in the intensive care unit in patients with ACS or stress cardiomyopathy. The most important characteristic of stress cardiomyopathy was its acute onset, and its typical clinical and radiological performance lasted for a short duration. Most studies obtained 2-dimensional echocardiography features of typical stress cardiomyopathy based on case reports: In the acute phase, the apical and middle segments of the left ventricle had abnormal ventricular wall motion, the basal segment had hyperactive ventricular wall motion coupled with apical ballooning as well as decreased LVEF and fractional shortening; and In the recovery phase, the left ventricular wall motion and LVEF returned to normal and the left ventricular extended and transient ventricular wall motion abnormalities occurred, resulting in decreased heart systolic function. The LVEF decreased significantly in the early stage of the disease, which gradually recovered within 1 week and recovered entirely to normal levels within 21 days. The left ventricular thrombosis could cause severe complications and adverse events of stress cardiomyopathy. Thus, the diagnosis of stress cardiomyopathy should be considered in patients with cardiogenic stroke, and left ventricular thrombosis usually occurs in the early stages of the disease.

## 6 Conclusion

Different patients with stress cardiomyopathy exhibited various clinical manifestations and characteristics, and echocardiography could aid in the early detection of the existence of left ventricular thrombus. Clinicians should be aware of the possible source of embolism in a subgroup of patients with a cerebral embolism. On the basis of the in-depth understanding of the syndrome of stress cardiomyopathy, clinicians should make the diagnosis early and develop a reasonable and effective therapeutic schedule to prevent the harmful effects of complications.

## Acknowledgment

All authors thank Dr. Samuel C. Dudley for reviewing and modifying this manuscript.

## Author contributions

**Conceptualization:** QY. Wang, H. Yu.

**Data curation:** H. Yu, C. Jiang, RM. Sun, HB. Cai, GL. Xu, F. Zhao, Q Kou.

**Formal analysis:** SG. Sun, ZC. Zhang

**Funding acquisition:** QY. Wang, SG Sun

**Supervision:** QY. Wang, J. Yu, F. Bai.

**Writing – original draft:** QY. Wang, H. Yu.

**Conceptualization:** Runmin Sun.

**Writing – original draft:** Qiongying Wang, Heng Yu, Cheng Jiang, Runmin Sun, Miaomiao Qi, Shougang Sun, Guangli Xu, Hongbin Cai, Zhenchang Zhang, Feng Zhao, Xiaoqing Kou, Jing Yu, Feng Bai.

**Writing – review & editing:** QY. Wang, F. Bai.
